# Abortion Case—Fatal Result

**Published:** 1857-08

**Authors:** 


*Abortion Case—Fatal Result.*

It seems that in July last a girl named Regnet Lawson was brought from Bureau County, Ill., to this city, accompanied by one Dr. Swanzey, for the purpose of concealment while an abortion could be induced. It appears that the object was accomplished by the doctor just named, who soon after left the girl in charge of others, and returned home. The operation, which was performed by instruments, was followed by uterine and peritoneal inflammation, and death on the 31st of July. Through information communicated by Dr. Bevan, who had been called to attend the poor girl, the Coroner summoned a jury of inquest, which, after a thorough and careful examination of witnesses, returned the following verdict:

"STATE OF ILLINOIS, COOK COUNTY, SS.

At an inquisition began at the city of Chicago, within the county of Cook, on the 1st day of August, and concluded on the 6th day of August, in the year one thousand eight hundred and fifty-seven, before George Hansen, Coroner of said county, upon view of the body of a dead woman there lying dead, by the oaths of S. D. Childs, etc. (jurors named), good and lawful men, who, being charged and sworn, to inquire for the State of Illinois where, how and by what means the said dead woman came to her death, upon their oaths do say that the said dead body has been identified and proved to be the dead body of Regnet Lawson, late of said Cook County, aged twenty-eight years; that she came to her death at said Chicago on the 31st day of July last, about the hour of nine of the clock in the forenoon of that day, from peritoneal inflammation or child-birth fever, caused by abortion, which has taken place about one or two weeks previous, and that said abortion was brought on and produced by the use of a certain instrument called a male catheter, and which said instrument was used by Dr. James Swanzey and James H. Temple, in and upon the body of the said Regnet Lawson, on or about the 15th day of July last, at said Chicago, in a manner tending to destroy the life of the said Regnet Lawson, with the intent then and there unlawfully and wilfully to cause and produce the abortion aforesaid, she, the said Regnet Lawson, then and there pregnant; and that an abortion of the fœtus of which she, the said Regnet Lawson, was then and there pregnant, was then and there caused and produced by the said Dr. Swanzey and James H.

Temple, in manner and by the means aforesaid, and that said abortion was the cause of the disease of which the body of the said Regnet Lawson came to its death, in manner and by the means aforesaid.

August 6, 1857.

Signed: S. D. Childs, foreman; W. F. Colby, W. W. Danenhower, Dolliver Walker, Julius Crone, J. M. Spears, Ernst Riedel, B. A. Stampoflskl, S. Beardley, W. F. Lavanway, William Yelverton and A. Gray.

The defendants, Dr. Swanzey and Mr. Temple, are still in custody, and are held on a Coroner's warrant. The next step will probably be an application for their release on a writ of habeas corpus."

The Mr. Temple named in the verdict is a resident of Bureau County, a widower, possessed of property, and hitherto bearing a respectable character. The girl had been living in his family, and was claimed to have become *ençiente* by him. The preliminary inquiry seems to have fully identified him as the principal actor, who had employed Dr. Swanzey, with the hope of concealing one most atrocious crime by committing another.

Personally we know nothing of either of the parties implicated in this affair; but, if proved guilty, we hope they will suffer the full penalties of the law. If guilty, both of them should spend the remainder of their days at hard work in the Penitentiary. We have no language to express our contempt of the man claiming to be a member of an honorable profession, who will lend himself to the work of embryotic murder, and if we are ever tempted to aid customers down stairs with the toe of our boot in close contact with the gluteal region, it is when they come to solicit aid to get rid of a fœtus in utero; and that, too, whether the applicant be male or female, married or unmarried. To ask a respectable physician to purposely produce abortion is as much an insult as to ask any other honorable citizen to commit a crime against the laws of God and man, for thirty pieces of silver. And the request should be as quickly and boldly resented. We make these remarks because many of our cotemporaries complain that such applications, even from married and respectable females, are amazingly frequent.

